# The relationship between challenge-hindrance stressors and AI dependence among art students: the mediating roles of creative self-efficacy and positive experiences

**DOI:** 10.3389/fpsyg.2026.1924924

**Published:** 2026-10-05

**Authors:** Huali Fan, Yuan Wang, Shengwu Pan, Shuai Shao

**Affiliations:** 1 School of Modern Media, Xinjiang Education Institute, Urumqi, Xinjiang, China; 2 School of Educational Science, Xinjiang Normal University, Urumqi, Xinjiang, China; 3 Xinjiang Key Laboratory of Education Cloud Technology and Resources, Urumqi, Xinjiang, China; 4 School of Mechanical and Electrical Engineering, Xinjiang Institute of Engineering, Urumqi, Xinjiang, China; 5 School of Information Engineering, Bayin Guoleng Vocational and Technical College, Korla, Xinjiang, China

**Keywords:** AI dependence, arts college students, challenge stressors, creative self-efficacy, hindrance stressors, I-PACE model, positive experiences

## Abstract

**Background:**

Despite growing concerns about art students’ over-reliance on generative AI tools, existing research has largely treated academic stress as a uniform construct, overlooking the possibility that different types of stressors may drive AI dependence through distinct psychological pathways.

**Methods:**

Based on the Interaction of Person-Affect-Cognition-Execution (I-PACE) model, this study examines the relationships between Challenge-Hindrance stressors (CHS) and AI dependence among art students. It explores the mediating roles of creative self-efficacy and positive experiences. A questionnaire survey was conducted among 580 art students using the Challenge-Hindrance Stress Scale, the Creative Self-Efficacy Scale, the Positive Experiences Scale, and the AI Dependence Scale.

**Results:**

Path analysis revealed that both challenge stressors (*β* = 0.245, *p* < 0.001) and hindrance stressors (*β* = 0.322, *p* < 0.001) were positively associated with AI dependence; challenge stressors were negatively associated with creative self-efficacy (*β* = −0.155, *p* = 0.003) and positively associated with positive experiences (*β* = 0.268, *p* < 0.001); the negative association between hindrance stressors and creative self-efficacy was significant (*β* = −0.107, *p* = 0.043). Mediation analysis indicated that creative self-efficacy played a significant mediating role only in the path involving challenge stressors (indirect effect = 0.023, 95% CI [0.007, 0.044]) and was not significant in the path involving hindrance stressors; positive experiences significantly served as a mediator in the relationships between both challenge stressors (indirect effect = 0.042, 95% CI [0.020, 0.068]) and hindrance stressors (indirect effect = 0.019, 95% CI [0.003, 0.039]) on AI dependence. The chain mediation effects did not reach significance in either path.

**Conclusion:**

The study found asymmetry in the dual pathways by which Challenge and Hindrance stressors relate to AI dependence. These findings extend the I-PACE model to the issue of AI dependence in arts education and provide empirical evidence and intervention insights for targeted prevention of problematic AI use.

## 1 Introduction

The rapid evolution and widespread adoption of generative artificial intelligence (AI) tools, such as Midjourney, Stable Diffusion, and ChatGPT, are profoundly reshaping creative practices in the fields of art and design (Anantrasirichai and Bull, 2021; Epstein and Hertzmann, 2023). These tools can quickly generate high-quality images, design variations, and creative concepts from text prompts, offering unprecedented convenience and efficiency for artistic creation (Rombach et al., 2022). However, their low barrier to entry and high output efficiency have also raised widespread concerns in the education sector: Will students’ over-reliance on AI hinder the development of their original thinking and independent creative abilities? Existing research indicates that AI dependence may lead to negative consequences, including declines in creativity, weakened critical thinking, misinformation, and increased cognitive laziness (Doshi and Hauser, 2024; Köbis et al., 2025).

Recent empirical research on the determinants of generative AI usage and dependence can be categorized into three broad clusters: technological factors, individual differences, and contextual factors (Cui and Han, 2026). From a technological perspective, scholars have focused on the ease of use, usability, and algorithmic transparency of AI tools, with findings suggesting that perceived usefulness and low cognitive friction are consistently associated with higher adoption and continued use (Klingbeil et al., 2024). From the individual-differences perspective, research has identified personality traits (Klingbeil et al., 2024), thinking habits (Schoeffer et al., 2025), and self-efficacy (Ye et al., 2024; Zhang and Xu, 2025) as significant predictors of AI reliance. From the contextual perspective, academic pressure (Liao et al., 2025; Zhang S. et al., 2024), time pressure (Abuzar et al., 2025), and task characteristics (Scharowski et al., 2023) have been found to be associated with students’ propensity to delegate creative work to AI.

Students at art colleges constitute a special group in research on AI dependence. Previous research has found that students majoring in fine arts report higher levels of psychological distress and stress than students in non-art majors, and they also devote more time to their studies (Lee et al., 2023; Zhang and Fah, 2025). First, art students face multifaceted pressures stemming from academic demands, career prospects, interpersonal relationships, emotions, and family (Abdi et al., 2010; Lee et al., 2023). The artistic creation process itself is characterized by high uncertainty, subjective evaluation, and self-involvement, which make students more prone to anxiety and cognitive load under creative pressure (Silvia and Kimbrel, 2010). Second, unlike academic tasks that can be objectively quantified, artistic creation centers on originality, aesthetic judgment, and personal expression (Pretz and McCollum, 2014). This means that the introduction of AI into the creative process may be associated with students’ most fundamental creative thinking and sense of identity. Third, the rapid penetration of generative AI tools into the field of art and design has presented art students with a constant challenge: AI-generated high-quality works continue to challenge their self-assessment of their own creative abilities (Epstein and Hertzmann, 2023). The complexity of art education may be linked to a greater susceptibility to AI dependence under academic pressure.

Despite these advances, three critical gaps remain. First, most existing studies treat academic stress as a homogeneous construct, relying on global measures of “stress” or “pressure” without distinguishing among qualitatively different types of stressors. This aggregation may obscure the distinct pathways through which growth-oriented demands versus obstructive barriers influence behavioral outcomes. Second, although the I-PACE model has been fruitfully applied to AI dependence (Ye et al., 2024; Zhang S. et al., 2024), no study to date has integrated the CHS framework into the I-PACE architecture to examine whether individual and affective mediators operate symmetrically or asymmetrically across stressor types. Third, the population of art and design students has received scant attention in the AI-dependence literature. Findings derived from general student samples may not generalize to creativity-intensive disciplines.

Thus, the present study addresses these gaps by situating the I-PACE model within the Challenge-Hindrance framework and testing its explanatory power specifically in arts education. Specifically, this study seeks to answer the following questions. How are challenge stressors and hindrance stressors associated with AI dependence through positive experiences and creative self-efficacy? Do these two types of stressors exert distinct effects through different psychological pathways?

## 2 Literature review

### 2.1 Theoretical foundation

The I-PACE model was used to describe and analyze individuals’ psychological and behavioral responses when faced with different situations (Brand et al., 2019). The model comprises four core components: (1) P-individual susceptibility (Person), such as personality traits, psychopathological tendencies, and biopsychological characteristics; (2) A-Affective response (Affect), referring to an individual’s emotional reactions to situational triggers and coping strategies; (3) C-Cognitive processing (Cognition), including maladaptive cognitions and cognitive biases; (4) E-Executive function (Execution), involving self-control, decision-making ability (Brand et al., 2019). The model emphasizes that addictive behavior does not stem from a single factor but from ongoing interactions among personal characteristics, emotional responses, cognitive processing, and executive behavior. Accordingly, addictive or problematic use can be understood as the behavioral outcome resulting from these interaction processes (Wang et al., 2025; Ye et al., 2024).

This I-PACE model has been successfully applied to areas such as online gaming disorder, social media addiction, and excessive smartphone use (Brand et al., 2019; Elhai et al., 2018; Wegmann et al., 2015). Recently, researchers have also begun applying it to issues of AI dependence. For example, Zhang S. et al. (2024) used the I-PACE model to explore the underlying relationships among academic self-efficacy, academic stress, performance expectations, and AI dependence; Ye et al. (2024) confirmed that inertial thinking predicts ChatGPT dependence through positive experiences and avoidance-oriented learning motivation. These studies indicate that the I-PACE model has good theoretical applicability for explaining AI dependence.

In this study, challenge and hindrance stressors are conceptualized as situational triggers. Existing research applying the I-PACE model has examined self-efficacy as a core construct within the personal factor (P) (Du et al., 2025; Zhang S. et al., 2024). Drawing on Ye et al. (2024), we locate positive experiences within the affective response component (A) of the I-PACE framework. Following the I-PACE framework, we conceptualize AI dependence as a problematic behavioral outcome resulting from impaired executive functions (Brand et al., 2019; Wang et al., 2025).

It should be noted that this study does not aim to validate the full I-PACE model; rather, it selectively applies the model, focusing on the core pathway P → A → E. The cognitive processing (C) component in the I-PACE model was not included as an independent variable in this study; this was not a theoretical oversight, but rather a deliberate choice based on the study’s focus. This selective approach is justified by the fact that the I-PACE model is a comprehensive framework for explaining the formation and maintenance of addictive behaviors (Brandtner et al., 2021; Wang and Wang, 2026), and different studies may test specific pathways within it depending on their research focus and data conditions (Brand et al., 2019; Ye et al., 2024).

### 2.2 Research hypotheses

#### 2.2.1 Challenge-hindrance stressors and AI dependence

Based on the intrinsic properties of pressure, stressors can be classified into challenge and hindrance stressors (Cavanaugh et al., 2000; Yao and Ma, 2021). Although challenge stressors and hindrance stressors both fall under the category of academic stress, they differ fundamentally in the pathways through which they affect an individual’s psychology and behavior.

Challenge stressors are demands that individuals perceive as requiring effort but contributing to personal growth, learning, and goal attainment, such as high workloads, time pressure, complex tasks, and a strong sense of responsibility (Yao et al., 2023). Artistic creation is a highly nonlinear, divergent cognitive activity that requires ample attentional resources for association, evaluation, and aesthetic judgment (Guilford, 1967; Runco and Jaeger, 2012). When challenge stress exceeds an individual’s cognitive load threshold, students’ attentional resources are heavily consumed by strategic thinking about how to complete the task on time, significantly reducing the space available for innovation and the expression of unique aesthetic sensibilities. Generative AI can quickly produce a variety of visual concepts, rapidly bridging the gap between technical execution and inspiration. Outsourcing labor-intensive execution to AI can boost creative efficiency in the short term and provide a strong sense of tool efficacy. However, according to behavioral reinforcement theory, once a positive feedback loop is established, students will turn to AI tools rather than draw on their own creativity when faced with challenging tasks. Over time, individuals’ reliance on AI evolves from “strategic borrowing” to “habitual substitution,” ultimately culminating in deep dependence, in which they are unable to initiate or complete the creative process without AI.

Hindrance stressors refer to demands that individuals perceive as hindering goal attainment, limiting personal development, and offering no value for growth, such as unreasonable course schedules and ambiguous evaluation criteria (Lepine et al., 2005; Podsakoff et al., 2007). According to the Stress-Coping Theory (Wills et al., 2001), stress may be associated with maladaptive coping behaviors. Hindrance stressors are more likely to elicit emotion-oriented or avoidance-oriented responses (Podsakoff et al., 2007; Zhang X. et al., 2024). In the context of art education, the harmful effects of hindrance stress are even more profound. The evaluation of artistic creation is inherently highly subjective. When students face unclear evaluation criteria or conflicting external expectations, they may experience the distressing sense that “no matter how hard I try, I may still fail to gain recognition.” This effort-reward discrepancy affects students’ intrinsic motivation for creation. Consequently, students may lose their anticipated control over both the creative process and its outcomes. According to Compensatory Control Theory (Kay et al., 2008; Landau et al., 2015), when individuals perceive a threat to their control over the external environment, they seek alternative sources of control to restore psychological balance. In the context of this study, students manipulate AI to achieve specific creative outcomes, thereby compensating for the loss of control in the real-world creative environment. This reliance does not stem from a pursuit of efficiency, but rather from a desire for psychological security. Each time students successfully use AI to circumvent obstacles and alleviate anxiety, the behavioral habit is reinforced through negative reinforcement. Compared with habits formed through positive reinforcement, dependency behaviors resulting from negative reinforcement are more intractable and harder to eliminate (Carter and Tiffany, 1999).

*H1a*: Challenge stressors are positively associated with AI dependence.

*H1b*: Hindrance stressors are positively associated with AI dependence.

#### 2.2.2 The mediating role of creative self-efficacy

Creative self-efficacy refers to an individual’s belief in their ability to generate novel and useful ideas (Tierney and Farmer, 2002). Challenge stressors, as a type of stress with growth-promoting attributes, typically elicit task-oriented coping in individuals. In such cases, students view AI as an efficient tool for enhancing creative efficiency and expanding creative boundaries; they proactively learn and master it to better complete tasks. This pattern of frequent, high-investment instrumental use serves as a crucial behavioral foundation for the development of AI dependence (Cavanaugh et al., 2000; Wills et al., 2001). However, unlike general academic tasks, artistic creation demands sustained originality, personal expression, and aesthetic judgment in every work. When students face high-intensity coursework and time pressure, they must make creative decisions rapidly; task difficulty can easily exceed their current creative capacity, leading to self-evaluations of creative inadequacy. Moreover, students repeatedly compare their own work to AI-generated outputs, which may erode their belief in their creative abilities. Students rely heavily on AI to meet deadlines, thereby exposing themselves to such upward comparisons more frequently. Therefore, in the specific context of arts education, we hypothesize that challenge stressors may be negatively associated with creative self-efficacy.

For hindrance stressors, the erosion of creative self-efficacy operates through resource depletion and learned helplessness. Hindrance stressors are generally regarded as negative factors that deplete individual psychological resources. When students face hindrance stressors such as unclear task requirements, lack of necessary resources for creation, or interpersonal conflicts in collaboration, their attentional resources are consumed by coping with the obstacles themselves. This continuously conveys a negative message that “I am not capable,” thereby constraining and undermining an individual’s creative self-confidence (Podsakoff et al., 2007; Yao and Yu, 2023). At the same time, persistently frustrating experiences may lead students to attribute difficulties in the creative process to a lack of ability, thereby undermining the development of their creative self-efficacy.

Regarding the behavioral outcomes of creative self-efficacy, it can significantly impact an individual’s technology use patterns. Individuals with high creative self-efficacy typically possess a more positive self-perception and a stronger sense of mastery over tools (Yao and Yu, 2023). When faced with difficulties and challenges in the creative process, they tend to view AI tools as extensions and levers of their creative abilities, confidently integrating them into their creative workflow to form a usage pattern grounded in confidence in their own abilities (Ye et al., 2024). When creative confidence is undermined, individuals are more likely to use AI as a tool to escape or relieve stress (Abbas et al., 2024; Zhang S. et al., 2024). Existing research indicates that individuals with low self-efficacy, when faced with tasks beyond their capabilities, are more likely to turn to AI for direct solutions, thereby exacerbating their dependence on this technology (Chen et al., 2026; Zhang and Xu, 2025; Zhang S. et al., 2024). Based on the above analysis, the following hypotheses are proposed:

*H2a*: Challenge stressors are negatively associated with creative self-efficacy.

*H2b*: Hindrance stressors are negatively associated with creative self-efficacy.

*H2c*: Creative self-efficacy is negatively associated with AI dependence.

*H2d*: Creative self-efficacy mediates the association between challenge stressors and AI dependence.

*H2e*: Creative self-efficacy mediates the association between hindrance stressors and AI dependence.

#### 2.2.3 The mediating role of positive experiences

Positive Experiences refer to the positive emotional experiences, such as pleasure, satisfaction, efficiency, and a sense of accomplishment that students gain when using AI tools for artistic creation (Ye et al., 2024). When students receive sustained positive emotional feedback from using AI tools, this experience reinforces their willingness to use AI, making them more inclined to use it repeatedly to obtain similar emotional rewards. As usage frequency increases and dependence deepens, students gradually develop emotional attachment and behavioral habits toward AI tools, ultimately leading to excessive dependence (Ye et al., 2024).

However, the nature and function of positive experiences differ markedly between the two pathways. For challenge stressors, positive experiences arise from authentic, growth-oriented engagement. Challenge stressors entail growth opportunities and an anticipated sense of accomplishment, which stimulate individuals’ engagement and positive emotional experiences (Cavanaugh et al., 2000; Sawhney and Michel, 2022; Zhang J. et al., 2024). When students perceive highly challenging creative tasks as worthwhile, they are more likely to enter a state of efficient, enjoyable creation with AI’s assistance. The rapid responsiveness and diverse outputs of AI tools can sustain and even amplify this positive experience. This positive emotional feedback reinforces students’ willingness to use AI tools, ultimately leading to dependence (Ye et al., 2024).

For hindrance stressors, positive experiences are largely compensatory and relief-oriented. Although hindrance stressors are typically viewed as negative factors that trigger negative emotions, their relationship with positive experiences may be more complex in the context of AI-assisted creation. According to stress coping theory, when individuals face uncontrollable difficulties, they may turn to external means to seek alternative sources of satisfaction and relief (Loureiro et al., 2023; Rodell and Judge, 2009). In artistic creation, when students face hindrance stressors, they may lower their task goals from “pursuing excellence” to merely “completing the task,” thereby reducing cognitive load. When AI tools help them quickly resolve hindrances, students experience positive emotional responses characterized by relaxation, reduced anxiety, and a temporary sense of progress (Peng et al., 2025; Yin et al., 2024). This compensatory, alternative positive experience may lead students to gradually view AI as an effective tool for rapidly alleviating hindrance stressors. Based on the above analysis, the following hypotheses are proposed:

*H3a*: Challenge stressors are positively associated with positive experiences.

*H3b*: Hindrance stressors are positively associated with positive experiences.

*H3c*: Positive experiences are positively associated with AI dependence.

*H3d*: Positive experiences mediate the association between challenge stressors and AI dependence.

*H3e*: Positive experiences mediate the association between hindrance stressors and AI dependence.

#### 2.2.4 The mediating role of creative self-efficacy and positive experiences

According to Control-Value Theory, individuals’ self-efficacy beliefs are directly related to their emotional responses (Pekrun, 2006). Individuals with high self-efficacy maintain favorable expectations for coping with difficulties; they are more likely to experience positive emotions, such as pleasure and pride, during tasks, whereas those with low self-efficacy tend to experience negative emotions, such as anxiety and frustration. Empirical research supports this view, showing that learners with higher self-efficacy experience positive engagement with AI tools and derive greater enjoyment from the process (Florescu et al., 2024; Mega et al., 2014). Following the general prediction of Control-Value Theory (Pekrun, 2006) and the I-PACE framework’s P → A pathway (Brand et al., 2019), we tentatively propose a positive CSE → PE relationship as the baseline hypothesis.

Within the framework of the I-PACE model, this implies a chain of effects: individual factors (creative self-efficacy) may be related to affective responses (positive experiences), which in turn shape behavioral outcomes (AI dependence) (Brand et al., 2019). Accordingly, we construct a mixed parallel-chain mediation model in which creative self-efficacy and positive experiences serve as both parallel mediators and chain mediators. Specifically, challenge and hindrance stressors are expected to be related to creative self-efficacy, which in turn shapes positive experiences, ultimately affecting AI dependence. In summary, this model is shown in Figure 1.

**Figure 1:**
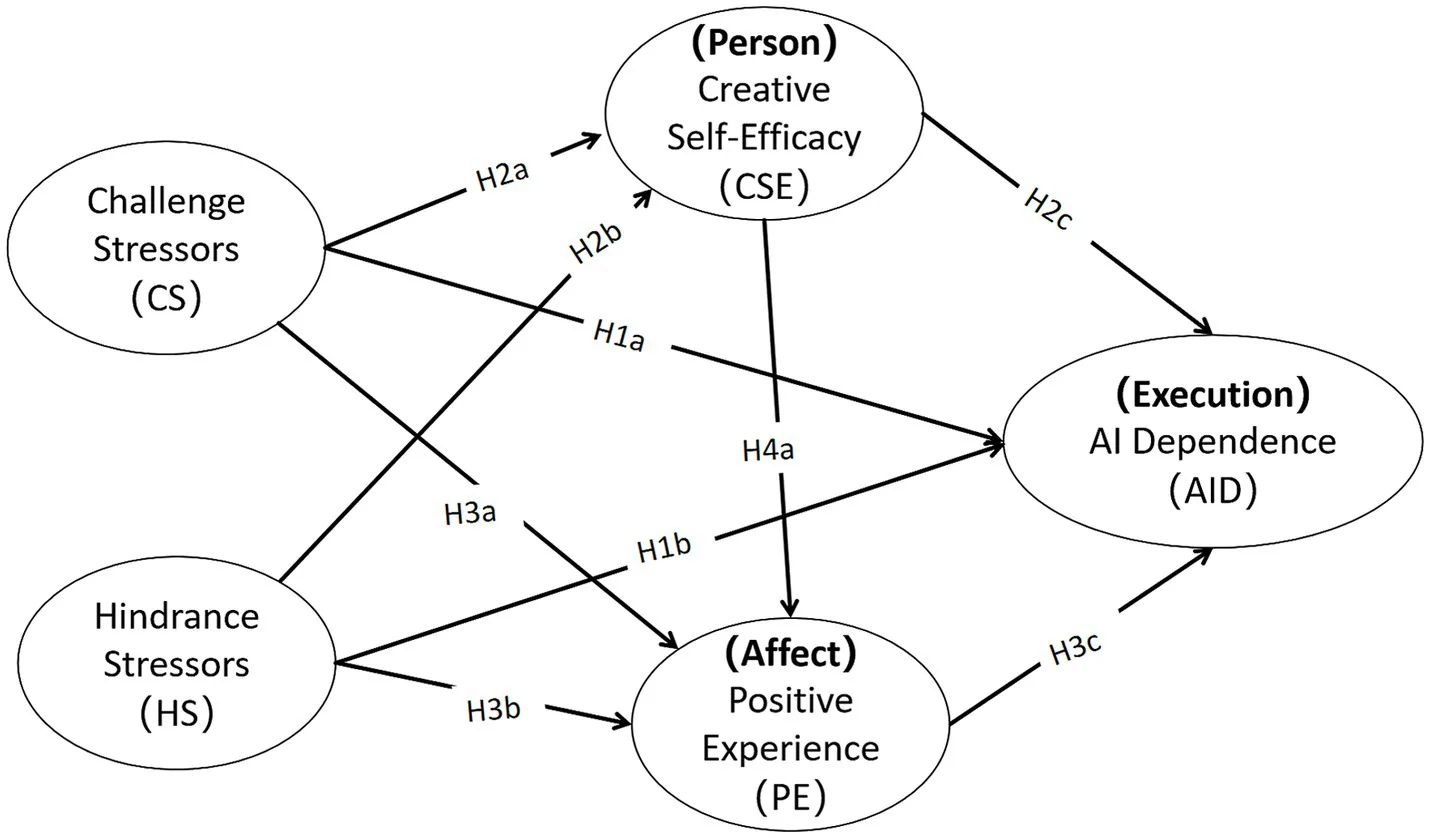
The hypothesis model. CS, challenge stressors; HS, hindrance stressors; CSE, creative self-efficacy; PE, positive experiences; AID, AI dependence (same applies below).

*H4a*: Creative self-efficacy is positively associated with positive experiences.

*H4b*: Creative self-efficacy and positive experiences play a chain-mediating role between challenge stressors and AI dependence.

*H4c*: Creative self-efficacy and positive experiences play a chain-mediating role between hindrance stressors and AI dependence.

## 3 Research design

### 3.1 Sample and data collection

This study employed purposive sampling to recruit undergraduate students majoring in art and design from multiple universities across various regions of China, including professional art academies, comprehensive universities, science and engineering universities, and teacher training universities. Data were collected from May to June 2026 through the “Wenjuanxing” online survey platform. A total of 620 questionnaires were distributed. After data collection, 40 responses were excluded based on the following criteria: (a) incomplete answers, (b) straight-lining responses, and (c) excessively short completion time (less than 120 s). The remaining 580 responses were retained for analysis, yielding a valid response rate of 93.5%. All participants were currently enrolled art or design majors and had prior experience using at least one generative AI tool for creative tasks. Participation was voluntary, and all respondents provided written informed consent before starting the survey. The final sample (*N* = 580) ranged in age from 18 to 25 years (M = 20.58, SD = 1.309). Demographic characteristics, including gender, grade, major, type of institution, AI experience, and weekly AI usage time, are summarized in Table 1. The sample of 580 participants substantially exceeds the recommended minimum of 200–400 for models of moderate complexity (Bentler and Chou, 1987), providing sufficient statistical power to detect the hypothesized mediation effects.

**Table 1:** Demographic information (*N* = 580).

Variable	Category	Number	Percentage	Variable	Category	Number	Percentage
Gender	Male	287	49.48%	Major	Visual Communication Design	73	12.59%
	Female	293	50.52%		Environmental Design/Interior Design	95	16.38%
Grade	Freshman	130	22.41%		Digital Media Art/Animation	96	16.55%
	Sophomore	195	33.62%		Fine Arts/Painting	75	12.93%
	Junior	164	28.28%		Product Design/Industrial Design	87	15.00%
	Senior	91	15.69%		Fashion and Apparel Design	54	9.31%
Types of higher education institutions	Professional Art Academies/Colleges of Art	163	28.10%		Photography/Visual Arts	45	7.76%
	Art Departments at Comprehensive Universities	238	41.03%		Sculpture/Public Art	26	4.48%
	Art Departments at Science and Engineering Universities	100	17.24%		Other	29	5.00%
	Art Departments at Teacher Training Universities	78	13.45%	Experience with AI	Less than 1 month	44	7.59%
	Other	1	0.17%		1–3 months	81	13.97%
Weekly AI usage time	Less than 1 h	84	14.48%		3–6 months	158	27.24%
	1–3 h	208	35.86%		6 months–1 year	203	35.00%
	3–5 h	167	28.79%		Over 1 year	94	16.21%
	5–10 h	93	16.03%				
	More than 10 h	28	4.83%				

This study strictly adhered to the ethical principles of the Declaration of Helsinki and was approved by the Ethics Committee of the Xinjiang Education Institute (protocol code XJEDU2026LLSC001, approved on 29 December 2025).

### 3.2 Measures

All scales used a 7-point Likert scale (1 = strongly disagree, 7 = strongly agree), with higher scores indicating a stronger manifestation of the corresponding trait. All scales were adapted following established guidelines for cross-cultural instrument adaptation (Brislin, 1970). The adaptation procedure included four steps: (a) the original English items were independently translated into Chinese by two bilingual researchers; discrepancies were resolved through discussion; (b) the Chinese version was back-translated into English by a third translator to verify conceptual equivalence; (c) the adapted scales were reviewed by three experts in educational psychology and arts education to assess content validity and relevance to the art student context; and (d) a pilot test was conducted with 50 art students (not included in the final sample) to assess item clarity and comprehensibility; minor wording adjustments were made based on their feedback. The final adapted scales demonstrated satisfactory content validity.

The Challenge-Hindrance stressors are based on those developed by Cavanaugh et al. (2000) and revised by Rodell and Judge (2009). We adapted the original items to the learning context of art college students by specifying “work” as “coursework and creative assignments,” “job” as “creative tasks,” and “employees” as “students.” Following the adaptation, the scale consists of 8 items for challenge stressors (example item: “I need to complete a large volume of coursework and creative assignments,” “I feel pressure due to time constraints,” “I need to apply a wide range of professional skills”) and 5 items for hindrance stressors (example item: “My advisor or instructor has presented me with conflicting expectations and requirements,” “The requirements for coursework and creative assignments are unclear,” “I lack the resources needed to complete creative assignments”).

The Positive Experience Scale was adapted from Ye et al. (2024). It was originally adapted from the Huang et al. (2020) value scale. The scale consists of 5 items that measure individuals’ perceived improvements in learning efficiency and positive experiences while using ChatGPT. To align with the artistic creation context of this study, the term “ChatGPT” in the items was uniformly replaced with “AI tools,” and “learning knowledge” was specified as “learning creation-related knowledge and skills.” The scale consists of 5 items. Sample items include: “Using AI tools makes it easier for me to learn creation-related knowledge and skills,” “AI tools help me master the knowledge required for creation more quickly,” and “AI tools are excellent partners in my creative learning.” Its items indeed measure cognitive appraisals and attitudinal tendencies, rather than pure momentary emotional states.

The Creative Self-Efficacy Scale was adopted from Tierney and Farmer (2002). It consists of 4 items and measures an individual’s belief in their ability to generate novel ideas.

The AI Dependence Scale was adopted from Zhang X. et al. (2025). It consists of 8 items and measures an individual’s level of dependence on AI tools. The scale has a unidimensional structure.

### 3.3 Data analysis

Descriptive statistics (means and standard deviations) and common method bias were assessed using SPSS 27.0. Confirmatory factor analysis (CFA) and path model were performed using AMOS 24.0. The maximum likelihood estimation method was used to estimate path coefficients, with model fit evaluated using multiple indices: χ^2^/df < 3, CFI > 0.90, TLI > 0.90, RMSEA < 0.08, and SRMR < 0.08. To test mediation effects, we employed the bias-corrected percentile bootstrap method with 5,000 resamples and reported standardized indirect effects with 95% confidence intervals.

Before the main analysis, we applied rigorous data-screening criteria, removing straight-lining responses and questionnaires with excessively short completion times. We confirmed that no significant outliers were present in the final sample (*N* = 580). Given the sample size and the use of bootstrap confidence intervals for all inferential tests, the potential impact of non-normality and minor extreme values on our substantive conclusions is substantially mitigated (Byrne, 2010).

## 4 Results

### 4.1 Common method bias

The common-method bias in the data was assessed using Harman’s one-factor test. An unrotated exploratory factor analysis was conducted on all measurement items. The results showed that the first factor had an eigenvalue of 9.857, explaining 32.856% of the variance, which is well below the critical threshold of 40%. Therefore, this study does not exhibit serious common method bias (Podsakoff et al., 2003).

### 4.2 Reliability and validity tests

The following sections present reliability and validity tests for the five variables: challenge stressors, hindrance stressors, creative self-efficacy, positive experiences, and AI dependence. As shown in Table 2, the Cronbach’s α coefficients for all variables are above 0.8, indicating that the scales possess good internal consistency reliability. In terms of convergent validity, the standardized factor loadings (λ) for the measurement indicators of each variable ranged from 0.602 to 0.869; the average variance extracted (AVE) values for challenge stressors, creative self-efficacy, positive experiences, and AI dependence were 0.521, 0.623, 0.595, and 0.548, respectively, all exceeding the recommended threshold of 0.5. The AVE value for hindrance stressors was 0.495, which, although slightly below 0.5, had a composite reliability (CR) above 0.7, and all factor loadings ranged from 0.602 to 0.798. Overall, it can still be considered to have acceptable convergent validity (Fornell and Larcker, 1981).

**Table 2:** Results of reliability and construct validity test.

Variable	Standardized Factor loadings (λ)	Cronbach’s α	AVE	CR
CS	0.685 ~ 0.775	0.896	0.521	0.897
HS	0.602 ~ 0.798	0.828	0.495	0.829
CSE	0.752 ~ 0.869	0.866	0.623	0.868
PE	0.727 ~ 0.797	0.878	0.595	0.880
AID	0.683 ~ 0.770	0.906	0.548	0.906

CS, Challenge Stressors; HS, Hindrance Stressors; CSE, Creative Self-Efficacy; PE, Positive Experiences; AID, AI Dependence (same applies below).

Discriminant validity was assessed by comparing the square roots of the AVE for each variable with the corresponding correlation coefficients. As shown in Table 3, the square root of the AVE for each latent variable was greater than the absolute value of the correlation coefficient between that variable and all other variables. The results indicate that the variables exhibit good discriminant validity (Fornell and Larcker, 1981). Discriminant validity was also verified using the heterotrait-monotrait ratio (HTMT) method; all HTMT ratios were below the strict threshold of 0.85, further supporting the discriminant validity among the variables.

**Table 3:** Results of the discriminant validity test.

Variable	CS	HS	CSE	PE	AID
Pearson Correlation and the Square Root of AVE (on the diagonal)					
CS	**0.722**				
HS	0.586	**0.704**			
CSE	−0.210	−0.189	**0.789**		
PE	0.347	0.285	−0.158	**0.771**	
AID	0.515	0.530	−0.288	0.356	**0.740**
HTMT					
CS	-				
HS	0.680	-			
CSE	0.238	0.224	-		
PE	0.393	0.333	0.182	-	
AID	0.574	0.613	0.324	0.400	-

The values on the diagonal are the square roots of AVE; the values in the lower triangle are Pearson correlation coefficients.

In summary, the reliability and validity of all variables in this study meet the basic requirements for empirical analysis. They can be used for subsequent path analysis.

### 4.3 Descriptive statistics and correlation analysis

The means, standard deviations, and Pearson correlation coefficients for each variable are shown in Table 4. Challenge stressors were significantly and positively correlated with AI dependence (*r* = 0.515, *p* < 0.01), significantly and negatively correlated with creative self-efficacy (*r* = −0.210, *p* < 0.01), and significantly and positively correlated with positive experiences (*r* = 0.347, *p* < 0.01). Hindrance stressors were significantly and positively correlated with AI dependence (*r* = 0.530, *p* < 0.01), significantly and negatively correlated with creative self-efficacy (*r* = −0.189, *p* < 0.01), and significantly and positively correlated with positive experiences (*r* = 0.285, *p* < 0.01). Creative self-efficacy was significantly negatively correlated with AI dependence (*r* = −0.288, *p* < 0.01), while positive experiences were significantly positively correlated with AI dependence (*r* = 0.356, *p* < 0.01). It is worth noting that creative self-efficacy was negatively correlated with positive experiences (*r* = −0.158, *p* < 0.01), which provides preliminary evidence for subsequent testing of chain mediation. The results of the correlation analysis provide preliminary support for the hypothesis testing.

**Table 4:** Means, standard deviations, and correlations among variables.

Variable	M	SD	CS	HS	CSE	PE	AID
CS	3.921	1.175	1				
HS	3.861	1.140	0.586**	1			
CSE	4.636	1.209	−0.210**	−0.189**	1		
PE	4.998	1.196	0.347**	0.285**	−0.158**	1	
AID	4.303	1.175	0.515**	0.530**	−0.288**	0.356**	1

**p < 0.01 (two-tailed test).

### 4.4 Path analysis

This study used AMOS 24.0 to conduct CFA on the measurement items for the five constructs: challenge stressors, hindrance stressors, creative self-efficacy, positive experiences, and AI dependence as shown in Table 5, the four-factor model (χ^2^/df = 3.496, CFI = 0.891, RMSEA = 0.066), the three-factor model (χ^2^/df = 7.001, CFI = 0.735, RMSEA = 0.102), and the two-factor model (χ^2^/df = 8.772, CFI = 0.655, RMSEA = 0.116), and the single-factor model (χ^2^/df = 11.047, CFI = 0.553, RMSEA = 0.132) all had significantly worse model fit than the five-factor model.

**Table 5:** Confirmatory factor analysis of the model (*N* = 580).

Models	χ^2^	df	χ^2^/df	CFI	TLI	RMSEA
Five-factor model: CS; HS; CSE; PE; AID	978.382	395	2.477	0.936	0.929	0.051
Four-factor model: CS + HS; CSE; PE; AID	1395.068	399	3.496	0.891	0.881	0.066
Three-factor model: CS + HS; CSE + PE; AID	2814.598	402	7.001	0.735	0.713	0.102
Two-factor model: CS + HS + CSE + PE; AID	3543.896	404	8.772	0.655	0.628	0.116
Single-factor model: All items combined	4473.864	405	11.047	0.553	0.520	0.132

“+” indicates that they are combined into a single factor.

The fit indices for the five-factor model were: χ^2^/df = 2.477, CFI = 0.936, TLI = 0.929, RMSEA = 0.051, SRMR = 0.042, GFI = 0.896, NFI = 0.897; all indices met or were close to acceptable standards. The single-factor model exhibited substantially poorer fit across all indices (χ^2^/df = 11.047, CFI = 0.553, TLI = 0.520, RMSEA = 0.132, SRMR = 0.095). The ΔCFI between the two models was 0.383, far exceeding the recommended threshold of 0.01 (Cheung and Rensvold, 2002), indicating that a single common factor cannot adequately account for the covariance among the observed variables. These findings provide strong evidence that common method bias does not pose a serious threat to the validity of our results. This result further corroborates the conclusion that common method bias does not seriously threaten the validity of our findings.

The R^2^ values for the endogenous variables were 0.137 for positive experiences, 0.051 for creative self-efficacy, and 0.392 for AI dependence, indicating that the model explained 13.7, 5.1, and 39.2% of the variances in these variables, respectively. To examine the hypothesized path relationships, we constructed a path model, and the standardized path coefficients are presented in Figure 2.

**Figure 2:**
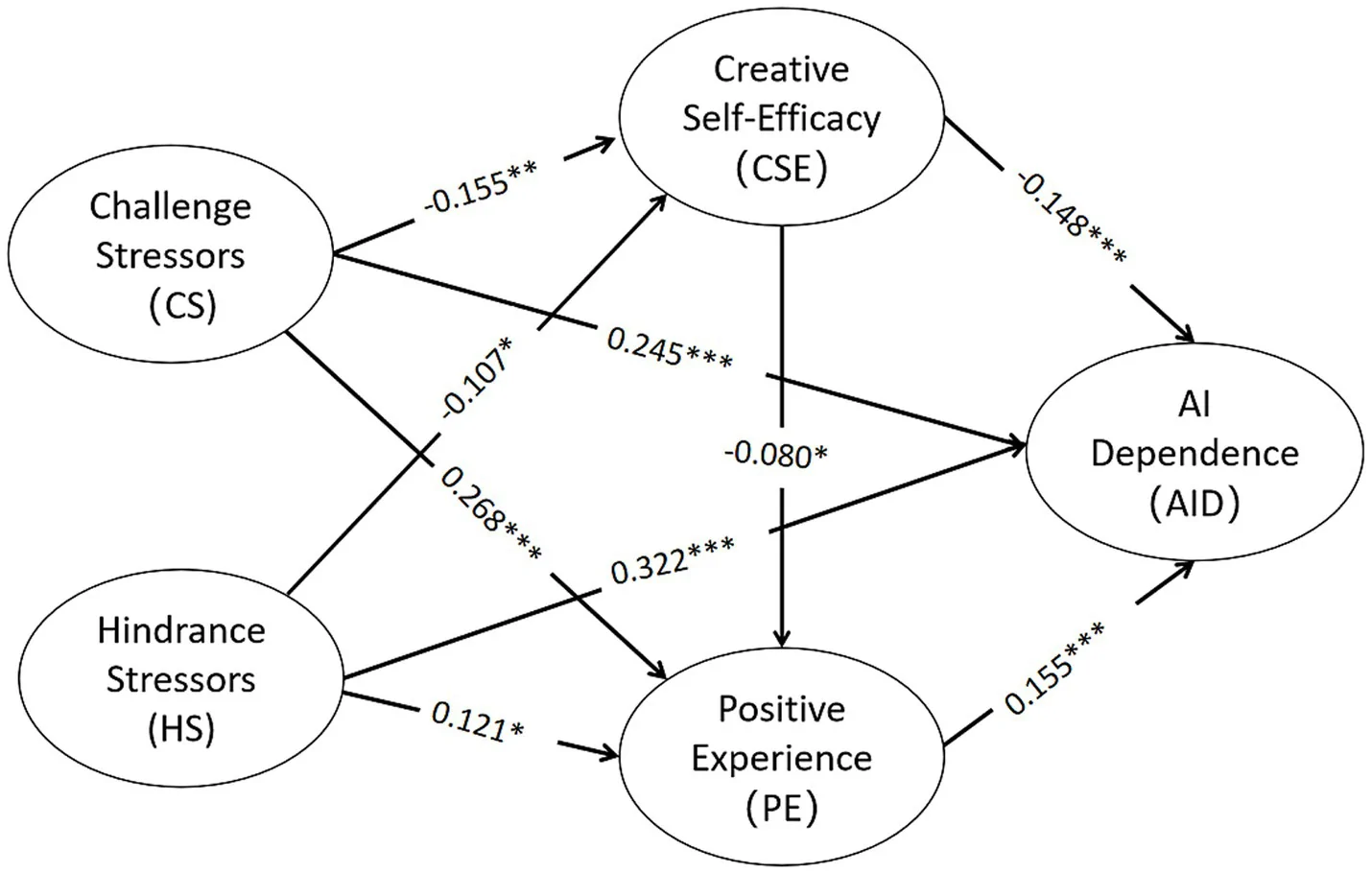
Model verification for challenge-hindrance stressors on AI dependence. * *p* < 0.05, ** *p* < 0.01, *** *p* < 0.001.

As shown in Figure 2, challenge stressors have a significant positive effect on AI dependence (*β* = 0.245, *p* < 0.001), and hindrance stressors also have a significant positive effect (*β* = 0.322, *p* < 0.001), confirming Hypotheses H1a and H1b.

Challenge stressors had a significant negative effect on creative self-efficacy (*β* = −0.155, *p* < 0.01), while hindrance stressors also had a significant negative effect (*β* = −0.107, *p* < 0.05), confirming Hypotheses H2a and H2b.

Challenge stressors had a significant positive effect on positive experiences (*β* = 0.268, *p* < 0.001), and hindrance stressors also had a significant positive effect (*β* = 0.121, *p* < 0.05), confirming Hypotheses H3a and H3b. The path from creative self-efficacy to positive experiences was significant but negative (*β* = −0.080, *p* < 0.05). Thus, Hypothesis H4a was not supported.

Finally, positive experiences had a significant positive effect on AI dependence (*β* = 0.155, *p* < 0.001), whereas creative self-efficacy had a significant negative effect (*β* = −0.148, *p* < 0.001). To further test the overall mediating effect, this study used the Bootstrap method to estimate the indirect effects with greater precision.

### 4.5 Bootstrap test of mediation effects

The Bootstrap method (5,000 repeated samples) was used to test the mediating effects of creative self-efficacy and positive experiences on the relationship between the two types of stressors and AI dependence. Given the significant correlation between challenge and hindrance stressors, we included both in the model to control for the impact of their shared variance on the path estimates. The test results are presented in Table 6.

**Table 6:** Results of the bootstrap test for mediating effects.

Effect type	Path	Effect size	Boot SE	95% CI	*p*	Results	Effect size (%)
Path of challenge stressors							
Direct effect	CS → AID	0.245	0.042	[0.164,0.327]	<0.001	Significant	78.53%
Mediating effect 1	CS → CSE → AID	0.023	0.010	[0.007, 0.044]	0.017	Significant	7.37%
Mediating effect 2	CS → PE → AID	0.042	0.012	[0.020, 0.068]	0.001	Significant	13.46%
Chain-based brokerage	CS → CSE → PE → AID	0.002	0.001	[0.000, 0.005]	0.128	Not significant	0.64%
Total indirect effects		0.067	0.015	[0.039,0.098]	<0.001	Significant	21.47%
Overall effect		0.312	0.042	[0.230,0.394]	<0.001	Significant	100%
Path of hindrance stressors							
Direct effect	HS → AID	0.322	0.042	[0.240,0.404]	<0.001	Significant	89.94%
Mediating effect 1	HS → CSE → AID	0.016	0.009	[0.000, 0.035]	0.070	Not Significant	4.47%
Mediating effect 2	HS → PE → AID	0.019	0.009	[0.003, 0.039]	0.044	Significant	5.31%
Chain-based brokerage	HS → CSE → PE → AID	0.001	0.001	[0.000, 0.004]	0.241	Not significant	0.28%
Total indirect effects		0.036	0.013	[0.013,0.064]	0.005	Significant	10.06%
Overall effect		0.358	0.043	[0.274,0.442]	<0.001	Significant	100%

*p < 0.05, **p < 0.01; All reported indirect effects are standardized (β); Bootstrap confidence intervals were estimated using the bias-corrected percentile method with 5,000 resamples. CI, confidence interval. If a Bootstrap confidence interval includes 0, it is considered non-significant.

The total effect of challenge stressors on AI dependence was significant (*β* = 0.312, *p* < 0.001), and the direct effect was also significant (*β* = 0.245, *p* < 0.001). Regarding indirect effects, the indirect effect of challenge stressors on AI dependence via creative self-efficacy was significant (effect size = 0.023, 95% CI [0.007, 0.044]), supporting H2d. The indirect effect via positive experiences was also significant (effect size = 0.042, 95% CI [0.020, 0.068]), supporting H3d. However, the chained mediation path (challenge stressors → creative self-efficacy → positive experiences → AI dependence) had an effect size of 0.002 with a 95% confidence interval of [0.000, 0.005], which included 0 (*p* = 0.128), and thus did not reach significance; therefore, H4b was not supported.

The overall effect of hindrance stressors on AI dependence was significant (β = 0.358, *p* < 0.001), and the direct effect was also significant (*β* = 0.322, *p* < 0.001). Regarding indirect effects, the indirect effect of hindrance stressors on AI dependence via creative self-efficacy was not significant (effect size = 0.016, 95% CI [0.000, 0.035], *p* = 0.070); thus, H2e was not supported. However, the indirect effect via positive experiences was significant (effect size = 0.019, 95% CI [0.003, 0.039]), supporting H3e. The chained mediation path (hindrance stressors → creative self-efficacy → positive experiences → AI dependence) had an effect size of 0.001 with a 95% CI of [−0.000, 0.004], which included 0, and was not significant; therefore, H4c was not supported.

## 5 Discussion

### 5.1 The direct relationship between challenge-hindrance stress and AI dependence

This study found that both challenge and hindrance stressors are positively associated with AI dependence. This finding contradicts the dichotomy in the classical stress framework that “challenge stress is beneficial, while hindrance stress is harmful” (Cavanaugh et al., 2000; Lepine et al., 2005) and highlights the unique role that AI tools play in creative education.

In this study, challenge stressors are positively associated with AI dependence, a finding that differs from the view proposed by Rodell and Judge (2009). This discrepancy may be related to differences in the research context and sample. Rodell and Judge (2009) focused on the stress responses of organizational members in the workplace, whereas this study focuses on a population of art students. For art students, stressors may be associated with the proactive use of AI tools to master the medium (Travis et al., 2020). When students face challenging, time-sensitive creative assignments, the intense workload may consume the cognitive resources needed for divergent thinking and in-depth aesthetic judgment. Students may turn to AI to quickly generate visual concepts, outsourcing the labor-intensive technical execution process and thereby potentially receiving immediate feedback on their progress. If such instrumental use were to become a conditioned reflex, it might contribute to a behavioral habit associated with dependence.

The finding in this study that hindrance stressors are positively associated with AI dependence is consistent with the findings of Cavanaugh et al. (2000) and Webster et al. (2011). When students realize that no matter how hard they try, their work may still be rejected due to subjective evaluation, AI tools may help them complete tasks more quickly, potentially alleviating their psychological burden.

Although both pathways are associated with dependence, the direct association of hindrance stressors (*β* = 0.322) is slightly stronger than that of challenge stressors (*β* = 0.245). This suggests that, compared to the pursuit of greater efficiency, avoiding uncertainty and seeking psychological safety may be more strongly linked to AI dependence. There are two possible explanations for this difference. Art and design courses are highly creative and open-ended. The challenge stressors that students simultaneously face include the demand to use generative AI tools proficiently. This finding provides empirical support for future interventions targeting hindrance stressors.

### 5.2 The mediating effects of creative self-efficacy

Creative self-efficacy significantly mediates the relationship between challenge stressors and AI dependence. This finding aligns with the conclusions of Zhang and Xu (2025) regarding the negative correlation between self-efficacy and technology dependence, as well as with those of Ye et al. (2024). However, its mediating effect was not significant in the path involving hindrance stressors. This result suggests that the mediating effect of creative self-efficacy is specific to the type of stressors.

The negative effect of challenge stressors differs from the classic conclusion that challenge stressors should enhance self-efficacy (Travis et al., 2020; Yao and Yu, 2023). A plausible interpretation is that when students face high-intensity coursework, complex creative tasks, and time pressures, they often must make quick decisions and produce work quickly. Under this pressure to produce at a high frequency, task difficulty may easily exceed students’ current creative ability, and students’ creative self-efficacy may be negatively affected by self-evaluations of creative inadequacy. When using AI tools, students are constantly exposed to high-quality, diverse creative works generated by AI. When students compare their own creative output, they might conclude that they are less creative than the AI, a perception that could be associated with lower creative self-confidence. This negative association is particularly evident in situations of challenge-induced pressure. When students are pressed for time and need to produce work quickly, they are more likely to rely on AI-generated content, which may expose them to upward comparison more frequently.

The non-significant mediating effect of creative self-efficacy in the hindrance stressor path warrants further reflection. One possible explanation is that hindrance stressors, by their very nature, are perceived as externally imposed and uncontrollable; students may attribute their creative difficulties not to personal inability. Such external attribution may protect creative self-efficacy from direct undermining, even though the stressors themselves are distressing.

### 5.3 The mediating effects of positive experiences

This study found that positive experiences mediate the associations between challenge stressors and hindrance stressors and AI dependence. Both types of stressors were associated with higher levels of AI dependence through their positive associations with positive experiences. However, fundamental differences in the pathways and mechanisms by which these two types of stressors relate to positive experiences point to distinct directions for practical interventions.

In the pathway involving challenge stressors, students view highly difficult creative tasks as challenges worth tackling (Lepine et al., 2005). When AI tools are perceived as effective in helping them overcome these challenges, students tend to report a sense of efficiency, control, and accomplishment during use. According to flow theory, when task difficulty is balanced with an individual’s skills, the individual is more likely to enter a state of flow (Huang and Liu, 2026; Zhang R. et al., 2025). This finding aligns with the theoretical predictions of the I-PACE model. It is consistent with the research conclusions of Ye et al. (2024), which found a positive correlation between positive AI usage and AI dependence. AI tools may be perceived as helping students maintain or enhance their creative efficacy when facing highly challenging tasks, thereby generating sustained positive experiences. This positive emotional feedback may, over time, be associated with increased willingness to use AI tools, which could contribute to a pattern of dependence (Triangga et al., 2026; Ye et al., 2024).

Hindrance stressors also showed an indirect association with AI dependence via positive experiences. The finding runs counter to the expectation that such stressors would trigger negative emotions. However, a deeper analysis reveals the underlying logic. Bandura (1997) points out that when individuals face uncontrollable predicaments, they may turn to external means to seek alternative sources of satisfaction. In artistic creation, hindering factors are common in the creative process (Silvia and Kimbrel, 2010). Students may turn to AI as an accessible external resource to regain a sense of control over the task, thereby obtaining immediate relief from the aversive state. When students use AI, these tools may rapidly generate alternative options, offer creative inspiration, or handle some of the foundational work, which might alleviate students’ anxiety and frustration in the short term and giving them a sense of relief (Ding et al., 2023). However, this positive experience is a compensatory substitute for satisfaction, masking stagnation or even regression in students’ creative abilities.

A comparison of the predictive strength of the two types of stressors for positive experiences reveals that challenge stressors are more strongly associated with positive experiences than hindrance stressors. This suggests that challenge stressors are better at eliciting authentic, growth-oriented positive experiences. In contrast, positive experiences associated with hindrance stressors largely stem from the fleeting sense of relief that follows problem-solving.

### 5.4 Chain mediation effects

This study showed that the chain transmission mechanism of “stressors → creative self-efficacy → positive experiences → AI dependence” did not reach statistical significance. A critical finding is that the effect of creative self-efficacy on positive experiences was significant and negative (*β* = −0.080, *p* < 0.05), which constitutes a key breakpoint that leads to the failure of the chained mediation. This negative relationship warrants closer examination. Drawing on expectancy disconfirmation theory (Oliver, 1980), this negative association suggests that students with high creative self-efficacy may experience a disconfirmation of expectations when confronted with the superior performance of AI-generated outputs. That is, their high confidence in their own creative abilities may set a high standard for comparison. Specifically, when juxtaposed with AI’s exceptional performance, this may be perceived as a threat to their creative identity. Moreover, from the perspective of cognitive dissonance theory, an alternative interpretation is that students who have invested substantial effort in developing their creative skills may perceive the ease and efficiency of AI-generated outputs as devaluing their hard-won abilities, a perception that may generate psychological discomfort and further reduce their positive experiences when using AI tools (Epstein and Hertzmann, 2023). This finding challenges the I-PACE model’s assumption that positive personal factors promote positive affect (Brand et al., 2019), suggesting a non-linear relationship between domain-specific self-efficacy and AI-related affective responses. It may be moderated by changes in reference standards and perceived identity threat, and calls for more context-sensitive refinement of the P → A pathway in AI-saturated creative domains.

This finding may explain why the P → A → E pathway of the I-PACE model is interrupted at the chain transmission stage in this study, and this further suggests that in contexts where AI technology instantly satisfies creative needs, high levels of creative self-efficacy do not linearly translate into positive affect; rather, they may evoke complex emotional ambivalence due to shifts in reference points, meaning that creative self-efficacy and positive experiences may exhibit a more complex pattern of interaction. It should be noted that these interpretations remain exploratory and require further validation in future research.

## 6 Research contributions

### 6.1 Theoretical significance

First, this study makes a theoretical contribution by integrating the CHS framework with the I-PACE model. Previous research on AI dependence antecedents has largely treated academic stress as a unidimensional variable, overlooking the qualitatively distinct nature of different types of stressors (Zhang S. et al., 2024). By introducing the CHS distinction into the I-PACE framework, this study shows that challenge stressors versus hindrance stressors operate through distinct psychological pathways to be related to behavioral outcomes. This integration broadens the I-PACE model’s applicability to educational contexts in which stress is a pervasive yet heterogeneous phenomenon.

Second, this study reveals an asymmetric dual-pathway mechanism linking challenge and hindrance stressors to AI dependence. The findings demonstrate that creative self-efficacy serves as a significant mediator only in the challenge stressor pathway, whereas positive experiences mediate the effects of both types of stressors. This asymmetry challenges the assumption that cognitive and affective pathways operate symmetrically across different stressor types. Moreover, the study uncovers an unexpected finding: creative self-efficacy was negatively associated with positive experiences in the chain mediation model. This suggests that in AI-saturated creative domains, high creative self-efficacy may paradoxically dampen positive affective responses when AI tools dominate the creative process. This finding implies that the relationship between personal factors and affective responses may be more complex in technology-embedded professional domains than originally theorized.

Third, this study may reveal conditions for the I-PACE model by testing it in the context of arts education. Art students constitute a theoretically distinctive population because their professional identity and self-worth are centrally tied to creativity, originality, and personal expression (Pretz and McCollum, 2014). The finding reveals that task success may depend heavily on originality rather than effort and strategy; self-evaluation is inherently subjective and involves constant social comparison; and AI tools provide easily accessible reference points that trigger upward comparisons. Arts education provides a theoretically informed context that sharpens the I-PACE model’s applicability to specific professional and educational populations.

### 6.2 Implications for practice

The findings of this study offer several tentative implications for art education practice and the management of AI use. These suggestions are derived from the study’s associational findings and might be considered illustrative; they require future experimental validation before being recommended as formal guidelines.

First, stressors should be distinguished, and differentiated interventions should be considered in curriculum design and instructional management. It may be beneficial for educators to recognize the fundamental difference between challenge stress and hindrance stress. For challenge stressors, the focus of instructional management lies in appropriately setting the intensity and frequency of challenging goals to prevent them from exceeding students’ coping thresholds and thereby eroding their creative confidence. In curriculum design, progressive structuring of creative assignments may help students accumulate experiences of validated competence as they gradually overcome challenges. For hindrance stressors, one possible focus could be systematically identifying and reducing unnecessary obstacles. For instance, clarifying evaluation criteria, establishing effective communication channels between instructors and students to address conflicts over expectations, and ensuring access to basic creative resources may be associated with a lower likelihood that students will use AI tools as a coping mechanism.

Second, educators might also consider addressing the potential erosive effects of challenge stressors on students’ creative confidence. Given that creative self-efficacy was negatively associated with AI dependence, reinforcing students’ creative self-confidence through progressive independent training and recognition of creative achievements could be a promising avenue. For instance, institutions could explore differentiated AI-use strategies across grade levels: lower-grade students might benefit from guided exposure to develop basic proficiency, whereas upper-grade students could be encouraged to engage in more independent creation with gradually reduced AI assistance. However, because these suggestions are based on cross-sectional findings, the specific thresholds and transition points for such scaffolding approaches would need to be validated through future longitudinal or experimental research before implementation.

Third, we should approach the effects of positive experiences with caution. A key to educational intervention lies in guiding students to distinguish between two distinct types of positive experiences: the “pleasure of efficiency through human-AI collaboration” and the “sense of accomplishment from independent creation.” Regarding the use of AI in challenging situations, educators may focus on guiding students to maintain positive experiences while avoiding overreliance on it. In contrast, in hindrance-stress situations, the focus should be on fundamentally reducing hindrance stress rather than simply encouraging or restricting AI use. Course designs should balance AI-assisted and AI-free creative activities to ensure students have opportunities to derive intrinsic satisfaction from independent creative processes. This recommendation aligns with the positive psychological significance of creative activity itself. Recent research suggests that creativity is not merely a product of intrinsic motivation, but that the act of creation itself can foster broader psychological experiences (Wang and Liu, 2026). Therefore, sustaining students’ independent creative engagement is not only a strategy to curb AI dependence but also a vital pathway to cultivating their overall psychological well-being and sense of purpose. This distinction offers important insights for subsequent interventions.

Fourth, developing a departmental policy framework for AI use may be beneficial. A possible approach could be for art and design departments to consider establishing tiered guidelines for AI use across different course types and levels, avoiding a “one-size-fits-all” approach. Incorporating engagement in the creative process and originality of creative concepts into evaluation criteria may alleviate stress-induced reliance on AI that students experience in pursuit of perfection. For students with low creative self-efficacy, intervention approaches may focus on building a record of successful experiences. Starting with simple tasks to gradually build a sense of creative success may help rebuild creative confidence.

### 6.3 Limitations and future directions

This study has several limitations that warrant attention in future research.

First, the cross-sectional study design limits causal inferences. Although this study developed theoretical hypotheses based on the I-PACE model, cross-sectional data cannot completely rule out reverse causality or the role of third variables (Maxwell et al., 2011). Future research should adopt a longitudinal tracking design or an experimental study to rigorously examine the dynamic causal relationship between students’ perceived stress and their dependence on AI.

Second, the sample’s representativeness and cross-cultural generalizability are limited. The sample in this study was drawn entirely from art and design majors at Chinese universities; whether the findings apply to student populations in other countries or regions remains to be further verified. Across different cultural contexts, there may be significant differences in art education philosophies, AI usage norms, and students’ cognitive approaches to stress. Future research may test the psychological mechanisms revealed in this study using cross-cultural samples to establish the external validity of the findings.

Third, all variables were measured using self-report scales, which may be subject to social desirability bias and other self-report artifacts. Although both Harman’s single-factor test and CFA model comparisons indicated that common method bias was not a major concern in this study, the inherent limitations of self-report data remain, and some residual bias cannot be completely ruled out. Future research may incorporate data on creative performance from teacher evaluations and platform-recorded AI usage behavior to diversify data sources and enhance the study’s objectivity.

Fourth, this study focused on mediating mechanisms and did not examine moderating variables, such as personality traits, AI literacy levels, and creative self-identity, that may be related to the strength of these pathways. Future research could further explore how these factors moderate the pathways identified in this study to construct a more comprehensive, context-specific theoretical model.

Fifth, the distinction and refinement of AI dependence are insufficient. Treating AI dependence as a unidimensional construct in this study may not fully capture its complexity. Future research should develop more discriminant measurement tools to investigate whether different types of stressors are positively associated with different types of AI dependence, as well as the differentiated effects of various dependence types on students’ long-term creative development.

Sixth, our measure of Positive Experiences was adapted from Ye et al. (2024) and primarily captures cognitive appraisals and attitudes toward AI, rather than pure emotional responses. This deviates from the original ‘affect’ component in the I-PACE model. While this operationalization follows prior published work in this emerging field, future research may employ separate measures for cognitive and emotional components to test the full model more precisely.

## Data Availability

The original contributions presented in the study are included in the article/supplementary material, further inquiries can be directed to the corresponding authors.
